# Comparative Metabolomic Analysis Reveals the Varied Accumulations of Functional Nutrients in Differentially Pigmented Mung Bean Seeds

**DOI:** 10.3390/foods15173020

**Published:** 2026-08-27

**Authors:** Hubin Yan, Wei Gao, Huijun Zhu, Qian Wang, Qingting Hao, Jianjun Yan, Yaowen Zhang, Zeyan Zhang

**Affiliations:** 1 College of Agronomy, Shanxi Agricultural University, Taiyuan 030031, China; maikyhb@126.com (H.Y.); zhuzhu2005024@163.com (H.Z.); 15101537964@163.com (Q.W.); haoqingting@sxau.edu.cn (Q.H.); yanjianjunsx@163.com (J.Y.); zyw8118571@126.com (Y.Z.); 2 Houji Laboratory in Shanxi Province, Shanxi Agricultural University, Taigu 030801, China

**Keywords:** mung bean, seed coat color, metabolomic analysis, bioactive compounds, antioxidant capacity

## Abstract

Coat color is closely associated with the nutritional and functional properties of mung bean (*Vigna radiata* L.) seeds. However, the relationship between the coat color and efficiency in nutrients of mung bean seeds has not been clearly clarified. In this study, comparative metabolomic analysis, bioactive compounds’ content measurement, and antioxidant capacities’ (ABTS^+^, DPPH, FRAP) determinations were conducted on four distinct varieties with different seed coat colors (black: BL, yellow: YE, yellow-green: YG, green: GR). A total of 1544 metabolites were identified in these mung bean seeds, with amino acids and derivatives, flavonoids, phenolic acids, lipids, and terpenoids, as the top five classes with high relative signal abundance. BL was abundant in flavonoids (20.12%), flavonols, anthocyanins (with six anthocyanins accounting for 22.32–565.21-fold of that in other varieties), free fatty acids, and phenolic acid. YE accumulated more amino acids and derivatives (31.85%), isoflavones (with daidzein accounting for 1.97–9.34-fold of other varieties), flavones, and lignans/coumarins. Antioxidant capacity determination results showed that BL exhibited the strongest FRAP (*p* < 0.05), YG had the highest DPPH scavenging activity (*p* < 0.05), while YE showed numerically the highest ABTS^+^ scavenging capacity (*p* > 0.05). Notably, anthocyanin accumulation was positively correlated with FRAP, while isoflavone and amino acid accumulations were positively correlated with ABTS^+^ scavenging capacity. These findings suggested that the overall antioxidant activity of mung bean seeds was jointly regulated by multiple metabolite classes. This study elucidates seed metabolomic variations among mung bean varieties, providing insights for functional breeding and precision food development.

## 1. Introduction

Mung bean (*Vigna radiata L.*), one of the most important edible legumes, is a high-protein but low-fat crop with both medicinal and food application values. It is cultivated on over six million hectares across Asia, Africa, South America, and Australia [1,2,3], with global production exceeding three million metric tons annually. Mung bean seeds are nutritionally balanced, serving as an excellent source of protein, dietary fiber, vitamins, and amino acids [1]. Additionally, they contain various bioactive compounds, including polyphenols, flavonoids, minerals, polysaccharides, and polypeptides [4,5], which contribute to their functional properties beyond basic nutrition. In recent decades, these nutrients and bioactive compounds have been identified to be closely associated with numerous health benefits [6,7], including liver protection and antihypertensive [8,9,10], hypoglycemic and hypolipidemic effects and detoxification [11,12,13], anticancer [4,7,14], anti-melanogenesis [15], alleviating heat stress [16], and immunomodulatory activities [17], establishing mung bean as a popular functional food.

Seed coat color represents one of the most visually distinctive and agronomically important traits in mung bean, directly influencing consumer preference, market value, and nutritional quality. In China, consumers are most familiar with green-coated mung beans, which are widely used in traditional soups, desserts, and sprouting products. In contrast, yellow mung beans are predominantly consumed in the Philippines and India, where they are preferred for specific culinary applications such as dhal preparation. Mung beans also have other seed coat colors, including black, brown, and mottled patterns [18], each associated with specific genetic backgrounds and adaptive characteristics. Notably, colored seeds, particularly black and red varieties, typically exhibit higher antioxidant activity than colorless ones [19], primarily due to the accumulation of anthocyanins and other phenolic compounds in the seed coat. Studies have revealed substantial variations in nutrient and bioactive compounds compositions among different colored mung beans, suggesting that color phenotype serves as a reliable indicator of functional quality. For instance, black mung beans possess significantly higher total phenolic acid content and antioxidant activity than conventional green varieties [11]. However, the metabolic profile differences among mung beans of varying colors still need to be systematically characterized, particularly regarding the specific metabolite classes and metabolic pathways underlying these variations.

Metabolomics has emerged as a powerful approach for comprehensive characterization of plant metabolic phenotypes, enabling the simultaneous detection and quantification of hundreds to thousands of metabolites. Among available analytical platforms, ultra-high-performance liquid chromatography coupled with triple quadrupole mass spectrometry (UHPLC-QqQ-MS) and ultra-performance liquid chromatography–tandem mass spectrometry (UPLC-MS/MS) offer unparalleled advantages for metabolomics research, including high sensitivity, resolution, and throughput for detecting and quantifying diverse metabolite classes—such as amino acids, lipids, organic acids, and polyphenols—in a single analytical run. These techniques have been extensively applied in plant metabolite analysis across various crops and fruits [20,21], as well as in studying mung bean stress responses, germination processes, and quality formation [16,22,23]. Nevertheless, comparative metabolomics studies specifically focusing on different colored mung bean varieties remain limited [23], representing a significant knowledge gap in understanding the metabolic basis of seed coat color formation and its functional implications.

With increasing consumer demand for functional foods tailored to specific health needs—such as antioxidant-rich products for chronic disease prevention or high-protein varieties for nutritional enhancement—clarifying the metabolic characteristics of different colored mung beans has become essential to guide targeted breeding programs and optimize resource utilization. This study integrates widely targeted metabolomics with comprehensive physiological and biochemical analyses to systematically identify characteristic metabolites, elucidate key metabolic pathways, and establish their correlations with antioxidant activities across four mung bean varieties with distinct seed coat colors (black: BL, yellow: YE, yellow-green: YG, green: GR). Using UPLC-MS/MS-based untargeted metabolomics coupled with bioactive compounds (including chlorophyll, carotenoids, total flavonoids, total phenols and anthocyanin) quantification and antioxidant capacity assays (2,2-diphenyl-1-picrylhydrazyl (DPPH) and 2,2′-azino-bis (3-ethylbenzothiazoline-6-sulfonicacid) (ABTS^+^) scavenging ability, and ferric-reducing antioxidant power (FRAP)), we investigated the associations among seed color, metabolome composition, and antioxidant activity. These findings will not only reveal the metabolic basis of seed coat color formation in mung beans but also provide a basis for developing functional mung bean products.

## 2. Materials and Methods

### 2.1. Plant Materials

**Figure 1:**
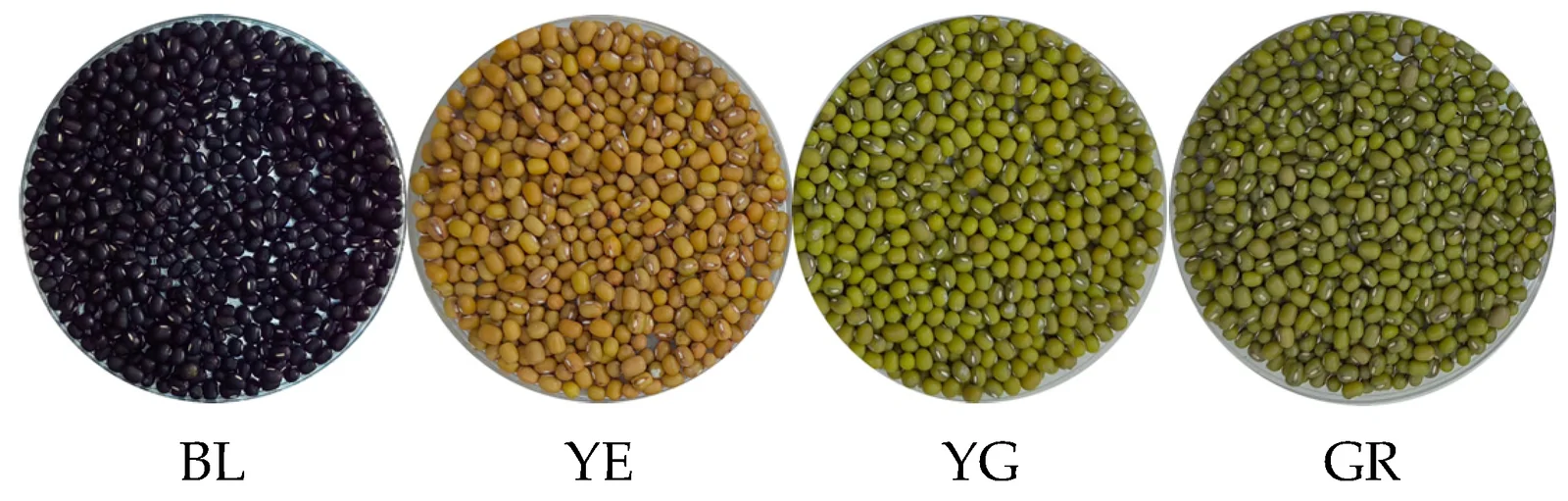
Seed morphology of four mung bean varieties with distinct seed coat colors. BL: black-seeded variety ‘Black Pearl’; YE: yellow-seeded variety ‘Zhonglü No. 16’; YG: yellow-green-seeded variety ‘Binglü No. 22’; GR: green-seeded variety ‘Jinlü No. 8’.

Seeds of ‘Black Pearl’ (with black seeds, BL), ‘Zhonglü No. 16’ (with yellow seeds, YE), ‘Binglü No. 22’ (with yellow-green seeds, YG), and ‘Jinlü No. 8’ (with green seeds, GR) exhibited distinct seed coat pigmentation phenotypes (Figure 1) and were harvested in August 2022 from the Dongyang Experimental Base of Shanxi Agricultural University, located in Yuci district, Jinzhong city, Shanxi province, China (112°40′51″ E, 37°33′05″ N). The black-seeded variety BL had a dark and uniform seed coat, YE presented a bright yellow seed coat, YG showed a yellow-green transitional seed coat color, and GR displayed a typical green seed coat phenotype. BL, YG, and GR are high-quality mung bean varieties bred by Shanxi Agricultural University (Shanxi, China), while YE was obtained from the Research Institute of Crop Science, Chinese Academy of Agricultural Sciences (Beijing, China).

### 2.2. Seed Color Parameter Measurement

By using a CR8 colorimeter (3nh, Guangzhou, China), color parameters of seeds of the four mung bean varieties were measured with ten replications [24].

### 2.3. Metabolites Extraction and Metabolomics Analysis

Mung bean seeds were lyophilized in a lyophilizer (Scientz-100F, Ningbo Scientz Biotechnology Co., Ltd., Ningbo, China) and ground into fine powders using a grinder (MM 400, Retsch GmbH, Haan, Germany). Subsequently, 50 mg of powder was extracted with 1200 μL of pre-cooled (−20 °C) 70% aqueous methanol containing internal standards. The mixture was vortexed for 30 s, repeated every 30 min for a total of six cycles, then centrifuged at 12,000 rpm for 3 min at 4 °C. The resulting supernatant was collected, filtered through a 0.22 μm microporous membrane, and transferred to an injection vial for UPLC-MS/MS analysis [25]. The UPLC-MS/MS-based widely targeted metabolomics sequencing is a relative quantification method. The obtained peak area reflects the relative abundance of metabolites.

### 2.4. Identification and Enrichment Analysis of Differentially Accumulated Metabolites (DAMs)

Detected metabolites were annotated using the KEGG COMPOUND database (http://www.kegg.jp/kegg/compound/, accessed on 10 March 2023) and subsequently mapped to the KEGG PATHWAY database (http://www.kegg.jp/kegg/pathway.html, accessed on 10 March 2023). Using the criteria of variable importance in projection (VIP) > 1 and absolute log_2_-fold change (|log_2_FC|) ≥ 1.0, differentially accumulated metabolites (DAMs) in seeds among the four varieties were identified. Pathways enriched with DAMs were then subjected to metabolite set enrichment analysis (MSEA), with significance determined by hypergeometric test (*p* < 0.05).

### 2.5. Determination of Chlorophyll and Carotenoids Contents

Chlorophyll and carotenoids extraction and determination were performed according to Gao et al. [26] and Zhang et al. [27], with modifications. Briefly, mung bean seeds were ground to powder in liquid nitrogen. Subsequently, 1 g of powder was extracted with 5 mL of acetone containing 0.1% (*w*/*v*) butylated hydroxytoluene (BHT), sonicated for 60 min, and centrifuged at 10,000 rpm for 15 min. The absorbance of the supernatant was measured at 663, 645, and 450 nm using a UV-1800 spectrophotometer (Shanghai Metash Instruments Co., Ltd., Shanghai, China) for chlorophyll and carotenoids quantification.

### 2.6. Determination of Total Flavonoids, Total Phenols, Anthocyanin, and Antioxidant Capacities

Mung bean seed powder (2.0 g) was mixed with 10 mL of 80% ethanol, vortexed, and ultrasonicated at 40 kHz for 15 min. The mixture was centrifuged at 5000 rpm at 4 °C for 10 min, and the supernatant was collected. The extraction was repeated twice, and the combined supernatants were diluted to 25 mL with 80% ethanol for subsequent analyses. Total flavonoid content and ABTS^+^ scavenging capacity were determined according to Fu et al. [28]. Total phenol content, DPPH scavenging capacity, and ferric-reducing antioxidant power (FRAP) were measured as described by Clarke et al. [29]. Trolox (≥98% purity, Sigma-Aldrich, St. Louis, MO, USA) was used as the reference standard for DPPH, ABTS^+^, and FRAP assays. Standard solutions with gradient concentrations (0–200 μmol/L for DPPH and ABTS^+^; 0–1000 μmol/L for FRAP) were prepared and assayed synchronously with samples in each batch to establish calibration curves. The antioxidant activities of samples were calculated based on the standard curves and expressed as mg Trolox equivalents (TE) per kg fresh weight (mg TE/kg FW). Standard controls were included in each assay plate to ensure quantification accuracy and batch-to-batch consistency. Anthocyanin extraction and determination were performed according to Zhuang et al. [30]. Briefly, 2.5 g of mung bean seeds powder was extracted with 25 mL of acidified ethanol (95% ethanol:1.5 M HCl = 85:15, *v*/*v*) for 24 h in the dark. Then, the absorbance at 535 nm was measured using a UV-1800 spectrophotometer (Shanghai Metash Instruments Co., Ltd., Shanghai, China).

### 2.7. Statistical Analysis

All data were represented as mean values ± standard deviation (SD, *n* ≥ 3) and subjected to one-way analysis of variance (ANOVA) based on a completely randomized design using SPSS 27.0 (SPSS Inc., Chicago, IL, USA). Principal component analysis (PCA) was conducted to explore metabolomic variation and relationships among metabolite profiles and antioxidant capacities [31] using OriginPro 2021 (OriginLab Corporation, Northampton, MA, USA). Redundancy analysis (RDA) was conducted using Canoco (Version 5.0, Microcomputer Power Corporation, Ithaca, NY, USA) to quantify the influences of metabolite classes, bioactive compounds, and color parameters on antioxidant activities. The significance of the RDA model was tested by 999 permutation tests.

## 3. Results

### 3.1. Metabolome Analysis Results of Differentially Pigmented Mung Bean Seeds

**Figure 2:**
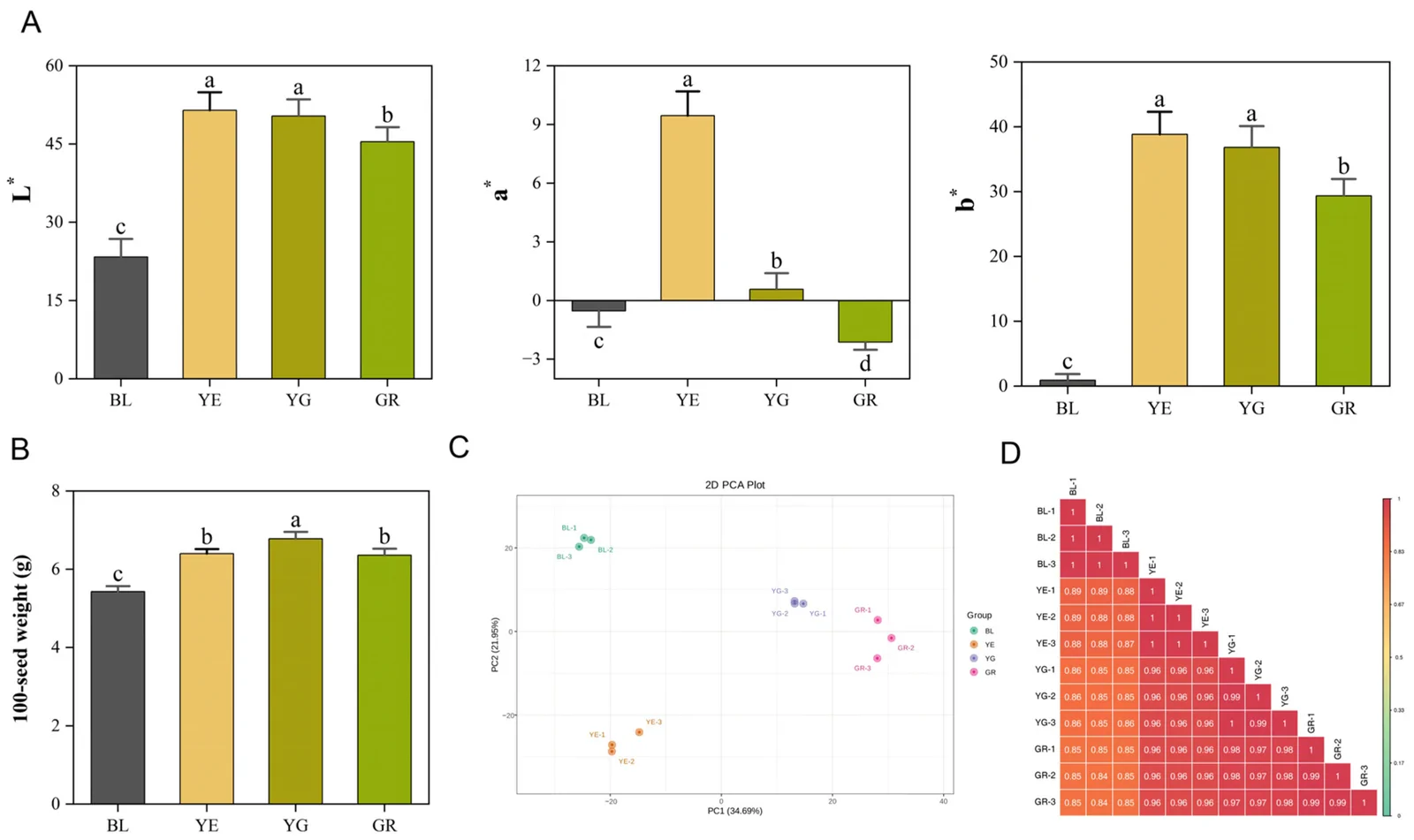
Phenotypic characterization and metabolic profiling comparisons of the four mung bean varieties. (**A**) L*, a*, b* color parameters. L* represents lightness, a* represents red–green chromaticity (positive = red, negative = green), and b* represents yellow–blue chromaticity (positive = yellow, negative = blue). (**B**) 100-seed weight. Data are presented as mean ± standard deviation (*n* = 3). Different lowercase letters indicate significant differences among varieties (*p* < 0.05, one-way ANOVA followed by Duncan’s multiple range test). (**C**) Principal component analysis (PCA) of metabolomic profiles. (**D**) Pearson correlation coefficients among samples.

Seed coat color parameters of the four mung bean varieties were first characterized (Figure 2A). Results showed that the L* values of YE and YG were significantly higher than those of BL and GR, with BL exhibiting the lowest L* value. The a* value was highest in YE, followed by YG, BL, and GR. The b* values of YE and YG were significantly higher than those of GR and BL. Additionally, YG displayed the highest 100-seed weight and largest seed size, followed by YE, GR, and BL (Figure 2B).

Metabolomic profiles of the four varieties were analyzed using UPLC-MS/MS. PCA revealed distinct clustering of the four varieties, with BL and YE clearly separated from YG and GR, indicating metabolic differentiation associated with seed coat color (Figure 2C). High reproducibility was confirmed among biological replicates (*r* = 0.993–0.997) (Figure 2D).

### 3.2. Metabolome Composition and Metabolites Abundance Analysis

A total of 1544 metabolites were identified from seeds across the four mung bean varieties (Table S1). These metabolites could be categorized into 14 classes: amino acids and derivatives (301), flavonoids (299), phenolic acids (160), lipids (159), terpenoids (121), alkaloids (100), organic acids (88), saccharides (76), lignans and coumarins (68), nucleotides and derivatives (67), quinones (20), vitamins (13), tannins (8), and others (64). Notably, some metabolites were only detected in certain varieties. For example, two triterpene saponins were detected in only BL and YG, and anthocyanidin delphinidin-3-*O*-(6″-*O*-feruloyl) glucoside was detected in only BL and YE (with its content in BL accounting for approximately 146-fold of YE).

**Figure 3:**
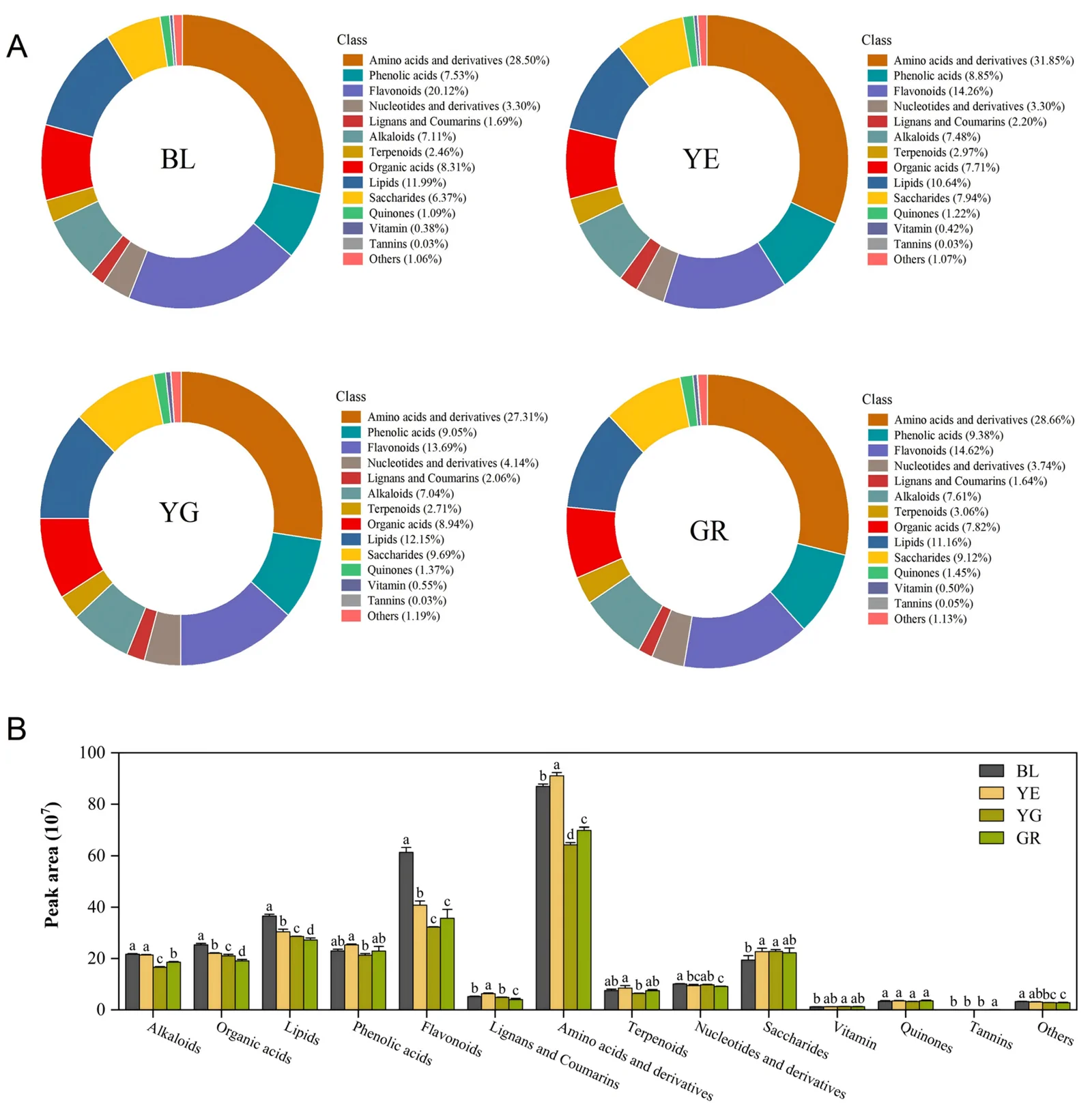
Metabolite compositions and relative abundances in seeds of the four mung bean varieties with distinct seed coat colors. (**A**) Circular diagram showing the relative proportion of major metabolite classes. Sector areas are proportional to metabolite class percentages. (**B**) Relative abundances of metabolite classes. Data are presented as mean ± standard deviation (*n* = 3). Different lowercase letters indicate significant differences among varieties (*p* < 0.05, one-way ANOVA followed by Duncan’s multiple range test).

The relative abundance of major metabolite classes varied significantly among the four mung bean varieties (Figure 3A). Among all varieties, YE had the highest relative signal proportion of amino acids and their derivatives, which accounted for 31.85% of the total metabolomic signal peak area. This was followed by GR (28.66%), BL (28.50%), and YG (27.31%). For flavonoids, BL showed the highest proportion at 20.12%, accounting for 1.47-, 1.41-, and 1.10-fold of that in YE, YG, and GR, respectively. GR had the highest relative abundance of phenolic acids at 9.38%, followed by YG (9.05%), YE (8.85%), and BL (7.53%). As for saccharides, their relative abundance ranked as follows: YG (9.69%) > GR (9.12%) > YE (7.71%) > BL (6.37%). A comparative analysis of primary metabolite relative contents (peak area-based) across colored mung bean varieties revealed substantial differences in relative signal abundance. BL had significantly higher flavonoid, lipid, and organic acid levels than YE, YG, and GR, with flavonoid content accounting for 1.5–1.9-fold of other groups. Both YE and BL also showed higher amino acid and derivative levels than YG and GR. Notably, lignans and coumarins were highest in YE, being 1.21-, 1.29-, and 1.56-fold of BL, YG, and GR, respectively (Figure 3B).

**Figure 4:**
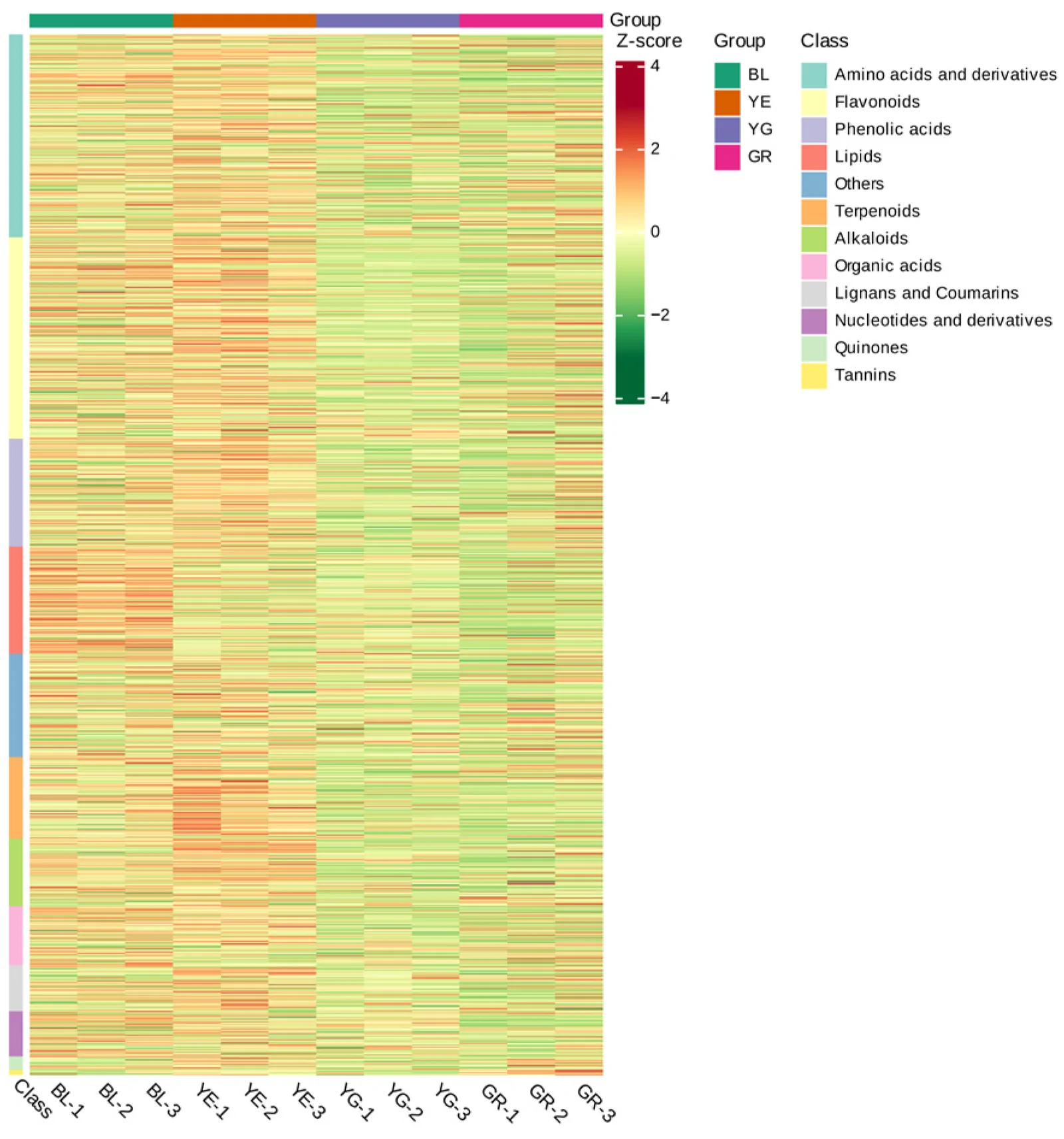
Hierarchical clustering heatmap of all detected metabolites. Colors represent normalized relative abundances (red, high; green, low).

Hierarchical cluster analysis (HCA) revealed distinct metabolite accumulation patterns among the four varieties (Figure 4). Amino acids and derivatives, flavonoids, and phenolic acids were more abundant in YE and BL than in YG and GR. Terpenoids, lignans, and coumarins showed higher accumulation in YE, whereas lipids and organic acids were most abundant in BL. Moreover, GR had the highest tannin abundance.

### 3.3. Identification of Differentially Accumulated Metabolites (DAMs)

**Figure 5:**
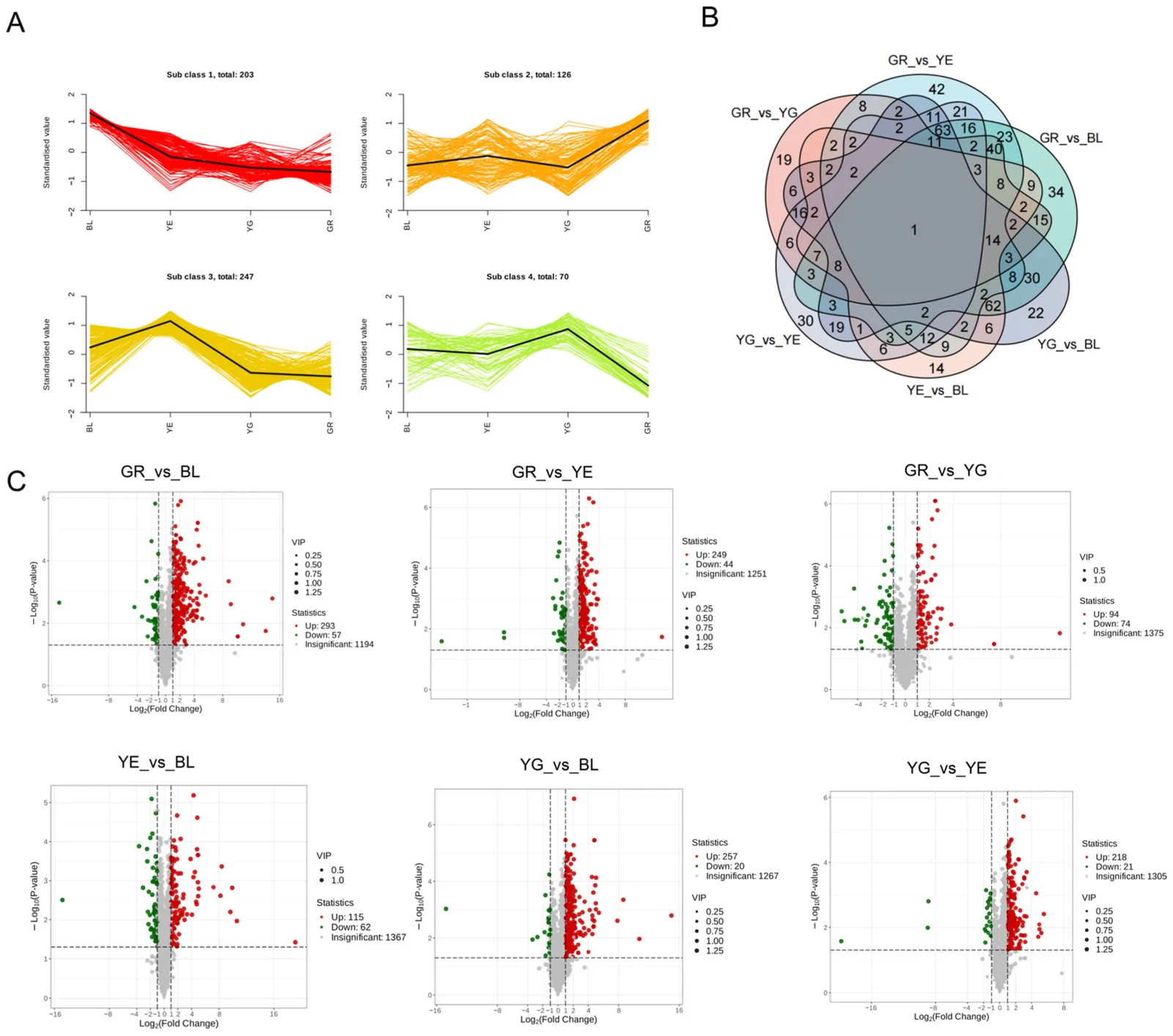
Identification and clustering analysis of differentially accumulated metabolites (DAMs). (**A**) K-means clustering analysis grouping 647 DAMs into four subclasses based on accumulation trends across BL, YE, YG, and GR. (**B**) Venn analysis results of DAMs from the six comparisons. (**C**) Volcano plots for the DAMs identified in each comparison. Red dot indicates significant upregulation, green dot indicates significant downregulation, and gray indicates no significant difference. The x-axis shows log_2_ (fold change, FC); the y-axis shows −log_10_ (*p*-value).

Using the criteria of |log_2_FC| ≥1 and VIP > 1, a total of 647 differentially accumulated metabolites (DAMs) were identified among the four mung bean varieties across six pairwise comparisons (Table S2). K-means clustering grouped these DAMs into four subclasses based on accumulation patterns (Figure 5A): Subclass 1 (203 DAMs, 31.4%): characterized by the highest accumulation in BL, enriched in flavonoids (32.0%) and lipids (20.7%); Subclass 2 (126 DAMs, 19.5%): showing the highest accumulation in GR, with amino acids and derivatives (27.8%) and flavonoids (19.8%) as major components; Subclass 3 (247 DAMs, 38.2%): exhibiting highest accumulation in YE, dominated by flavonoids (25.5%), amino acids and derivatives (17.4%), terpenoids (16.2%), and phenolic acids (14.2%); and Subclass 4 (71 DAMs, 11.0%): displaying highest accumulation in YG, primarily composed of amino acids and derivatives (24.3%).

Among the 647 DAMs, 34, 42, 19, 14, 22 and 30 metabolites were found to be specific in GR_vs_BL, GR_vs_YE, GR_vs_YG, YE_vs_BL, YG_vs_BL, and YG_vs_YE comparison respectively. These comparison-specific DAMs predominantly belonged to flavonoids, phenolic acids, and amino acid derivatives. For BL-centric comparisons (GR_vs_BL, YG_vs_BL and YE_vs_BL), 62 shared DAMs were identified, of which 59 (95.2%, including 20 free fatty acids, 13 flavonols, and 5 anthocyanidins) were upregulated in BL—consistent with BL’s enrichment in fatty acid and flavonoids subclasses. In contrast, YE-centric comparisons (GR_vs_YE, YE_vs_BL and YG_vs_YE) shared 12 DAMs, with 10 (83.3%) upregulated in YE, predominantly flavonoids (e.g., vitexin derivatives and isoflavones) (Figure 5B). Only one DAM, 1-methylxanthine, was shared across all six comparisons, with highest accumulation in YE, accounting for 4.42-, 2.02-, and 1.20-fold of BL, YG, and GR, respectively.

Across all six pairwise comparisons, the total number of DAMs varied from 168 (between GR and YG) to 350 (between GR and BL) (Figure 5C). Notably, BL and YE displayed a high proportion of upregulated DAMs in their respective comparisons. This finding indicates that BL and YE seeds may possess more abundant metabolite profiles than GR seeds.

### 3.4. KEGG Enrichment Analysis of DAMs

**Figure 6:**
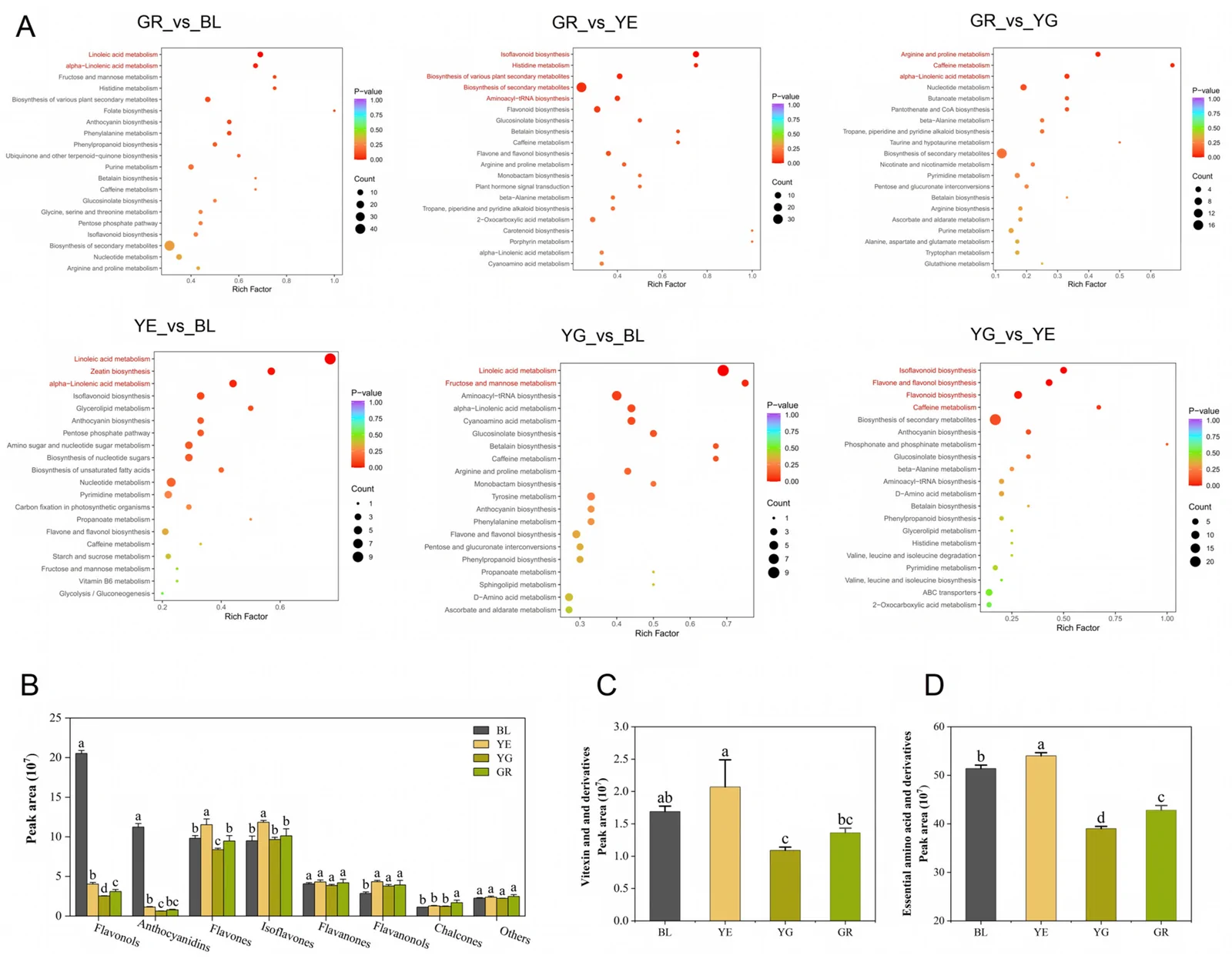
Metabolic pathway enrichment and metabolite quantification analysis results. (**A**) KEGG pathway enrichment bubble plots for six pairwise comparisons. Rich factor (x-axis) indicates the ratio of enriched DAMs to total background metabolites. Pathway names (y-axis) are shown in red when *p* < 0.05. Dot color gradient from blue to red indicates decreasing *p*-value; dot size is proportional to DAM count. (**B**) Flavonoid relative content. (**C**) Vitexin and derivative relative content. (**D**) Amino acid and derivative relative content. Each bar represents an individual metabolite. Data are presented as mean ± standard deviation (n = 3). Different lowercase letters indicate significant differences among varieties (*p* < 0.05, one-way ANOVA followed by Duncan’s multiple range test).

Metabolic pathway enrichment analysis was performed using the KEGG database [32,33]. DAMs from the six pairwise comparisons (GR_vs_BL, YE_vs_BL, YG_vs_BL, GR_vs_YE, YG_vs_YE and GR_vs_YG) enriched 70, 39, 62, 66, 47, and 43 pathways (Figure 6A), respectively.

DAMs of BL-centric comparisons (GR_vs_BL, YG_vs_BL and YE_vs_BL) showed significant enrichment in fatty acid metabolism (linoleic acid metabolism, ko00591; α-linolenic acid metabolism, ko00592) and flavonoid biosynthesis (anthocyanin biosynthesis, ko00942; flavone and flavonol biosynthesis, ko00944) pathways. Notably, free fatty acid-related DAMs in BL were specifically enriched in linoleic and α-linolenic acid metabolism. YE-centric comparisons (GR_vs_YE, YG_vs_YE and YE_vs_BL) were enriched in isoflavonoid biosynthesis (ko00943), general flavonoid biosynthesis (ko00941), and histidine metabolism (ko00340) pathways. Isoflavone-related DAMs in YE were uniquely enriched in the isoflavonoid biosynthesis pathway. GR- and YG-centric comparisons showed enrichment in caffeine metabolism (ko00232, the only pathway enriched by DAMs of all six comparisons) and arginine and proline metabolism (ko00330) pathways. Shared pathways across specific comparisons included phenylalanine metabolism, phenylpropanoid biosynthesis, and betalain biosynthesis (all DAMs upregulated in GR/YG_vs_BL comparisons), as well as secondary metabolite biosynthesis and aminoacyl-tRNA biosynthesis (most DAMs upregulated in YE in GR/YG_vs_YE comparisons) pathways. Anthocyanidin biosynthesis pathway was specifically enriched in BL_vs_GR/YG/YE comparisons, while flavone/flavonol biosynthesis pathway was consistently enriched in both BL_vs_YG/YE and YE_vs_GR/YG comparisons (Table S7).

### 3.5. Main Bioactive Differentially Accumulated Metabolites Among the Four Mung Bean Seeds

To further elucidate the relative abundance and bioactive functions of key metabolites, we focused on major mung bean constituents—including polyphenols (flavonoids and phenolic acids), free fatty acids, and amino acids—based on the comprehensive metabolite profiling and DAM analysis.

#### 3.5.1. Flavonoids

A total of 299 flavonoid metabolites were identified, comprising 89 flavones, 69 flavonols, 34 flavanones, 26 isoflavones, 22 anthocyanidins, 17 other flavonoids, 16 chalcones, 16 flavanols, and 10 flavanonols. Isoflavones and flavones were most abundant in YE, with significantly higher levels compared to BL, YG, and GR. However, flavonols and anthocyanidins were markedly enriched in BL, exhibiting 5.10-, 8.16-, and 6.63-fold (for flavonols) and 9.96-, 18.06-, and 14.55-fold (for anthocyanidins) of that in YE, YG, and GR, respectively (Figure 6B).

Isoflavone DAMs (YE_vs_BL/YG/GR) were enriched in isoflavonoid biosynthesis. Prunetin, glycitin, 6″-*O*-Malonyldaidzin, daidzin and daidzein were 1.97- to 9.34-fold higher in YE. Three anthocyanins (kuromanin, delphinidin-3-*O*-(6″-*O*-p-coumaroyl)glucoside, mirtillin) annotated in the anthocyanin biosynthesis pathway, along with two anthocyanidins (delphinidin-3-*O*-galactoside and cyanidin-3-*O*-galactoside) unannotated in this pathway, showed a 22.32- to 565.21-fold increase in BL. Additionally, delphinidin-3-*O*-(6″-*O*-feruloyl) glucoside was exclusively detected in BL and YE, with its content in BL being 146.05-fold higher than that in YE. Flavonol DAMs (BL_vs_YE/YG/GR) were enriched in flavone and flavonol biosynthesis. Rutin, baimaside, and seven unannotated flavonols were 2.54- to 33.0-fold higher in BL. Flavone DAMs (YE_vs_others) were enriched in the same pathway, with acacetin 2.25- to 4.24-fold higher in YE. YE exhibited the highest vitexin and isovitexin derivative content (1.22- to 1.90-fold higher than others; Figure 6C), with vitexin-2″-*O*-rhamnoside (VOR) and isovitexin-2″-*O*-rhamnoside as major components. Conversely, vitexilactone was highest in BL (3.38- to 5.03-fold of other varieties) (Table S3).

#### 3.5.2. Free Fatty Acids

KEGG enrichment analysis demonstrated that the DAMs between BL and YE/YG/GR were significantly enriched in the linoleic acid metabolism pathway. Compared to YE, YG, and GR, nine free fatty acids, including TriHOME and EpOME, were 3.11- to 20.07-fold higher in BL. Additionally, four DAMs were significantly enriched in the α-linolenic acid metabolism pathway, being 2.08- to 6.01-fold of YE, YG, and GR in BL (Table S4).

#### 3.5.3. Phenolic Acids

Thirty-three free phenolic acids were detected, with benzoylmalic acid, 2-*O*-caffeoylglucaric acid, 4-hydroxybenzoic acid, 2-hydroxycinnamic acid, and α-hydroxycinnamic acid as the top five. Free phenolic acids constituted 23.61% of total phenolic acids in BL, which was significantly higher than that in YE (19.46%), YG (18.46%), and GR (18.27%) (Table S5).

#### 3.5.4. Amino Acids and Derivatives

All 20 proteinogenic amino acids and di-to pentapeptides were detected in mung bean seeds. Of them, L-phenylalanine, L-histidine, L-tryptophan, L-valine, L-glutamic acid, and L-arginine were abundant across all varieties. L-histidine, L-valine, L-arginine, and L-methionine were significantly abundant in BL and YE versus YG and GR (Table S6). Essential amino acids and derivatives content was the highest in YE, accounting for 1.06-, 1.39-, and 1.24-fold of BL, YG, and GR (Figure 6D), respectively.

### 3.6. Antioxidant Activities and Bioactive Compounds Contents Determination

**Figure 7:**
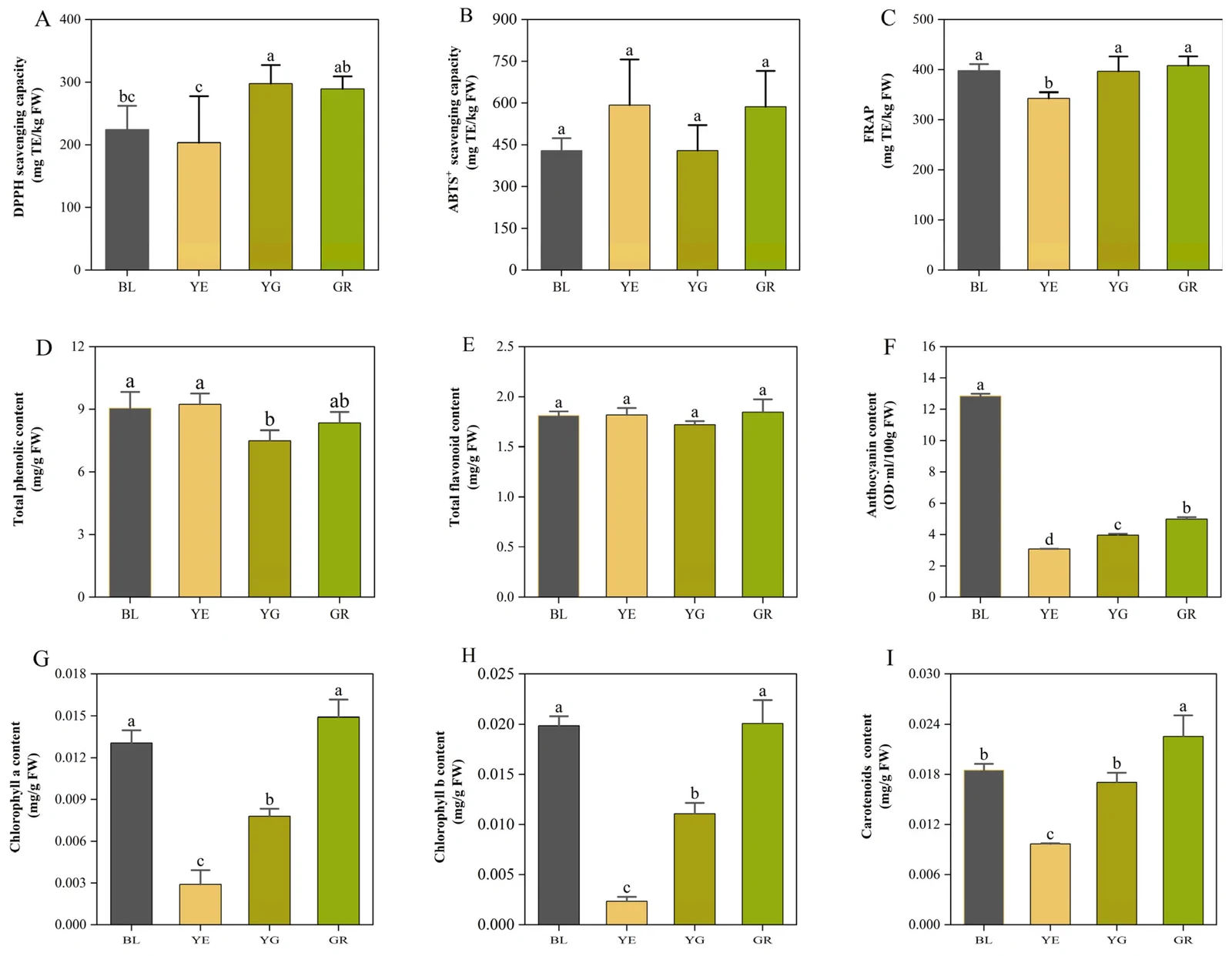
Physiological and biochemical indices of four mung bean varieties with distinct seed coat colors. (**A**) DPPH (2,2-diphenyl-1-picrylhydrazyl) scavenging capacity. (**B**) ABTS^+^ (2,2′-azino-bis(3-ethylbenzothiazoline-6-sulfonic acid)) scavenging capacity. (**C**) FRAP (ferric ion reducing antioxidant power). (**D**) Total phenolic content. (**E**) Total flavonoid content. (**F**) Anthocyanin content. (**G**) Chlorophyll a content. (**H**) Chlorophyll b content. (**I**) Carotenoids content. All antioxidant activity assays were performed with Trolox as the reference standard, and results are expressed as Trolox equivalents. FW: fresh weight. Different lowercase letters above columns indicate significant differences among varieties (*p* < 0.05).

Mung beans are rich in secondary metabolites such as flavonoids and phenolic acids, which significantly contribute to human health, particularly through their antioxidant activity, making them an ideal source of natural antioxidants. Mung bean extracts were evaluated for antioxidant activity using DPPH, ABTS^+^, and FRAP assays (Figure 7A–C). YG exhibited the highest DPPH scavenging activity (297.7 ± 29.7 mg TE/kg FW), significantly exceeding BL and YE (*p* < 0.05). YE showed the numerically highest ABTS^+^ scavenging capacity (592.4 mg TE/kg FW), followed by GR, BL, and YG, with values ranging from 428.9 to 547.2 mg TE/kg FW for the other three varieties. However, no statistically significant difference was observed in ABTS^+^ scavenging capacity among the four varieties (*p* > 0.05). BL displayed the strongest FRAP reducing power (398.1 mg TE/kg FW), while YE showed the lowest (342.5 ± 12.2 mg TE/kg FW, *p* < 0.05). These method-dependent variations reflect differences in assay reaction mechanisms rather than absolute antioxidant capacity.

The contents of bioactive substances in mung beans of the four seed color varieties were measured. Total phenolic content differed significantly among the four mung bean varieties: the total phenolic content in YE (9.24 mg/g FW) and BL (9.06 mg/g FW) ranked top two, and was higher than GR (8.35 mg/g FW), but significantly higher than YG (7.49 mg/g FW) (Figure 7D). Total flavonoid content, as determined by physiological analysis, showed no significant variation across varieties (Figure 7E), a finding that different from the metabolomic profiling, which may be attributed to differences in detection sensitivity or the contribution of minor flavonoid subclasses that were not captured in the metabolomic profiling. BL exhibited the highest anthocyanin content, which was 4.17-, 3.24-, and 2.58-fold higher than that in YE, YG, and GR, respectively (Figure 7F). This result was consistent with the metabolomic data in terms of change trend, but the fold-change magnitudes differed between the two methods.

The chlorophyll a and b contents were highest in BL and GR, intermediate in YG, and lowest in YE (Figure 7G,H). Carotenoids were the most abundant in GR, accounting for 1.22-, 1.32-, and 2.33-fold of BL, YG and YE (Figure 7I), respectively.

### 3.7. PCA and RDA

**Figure 8:**
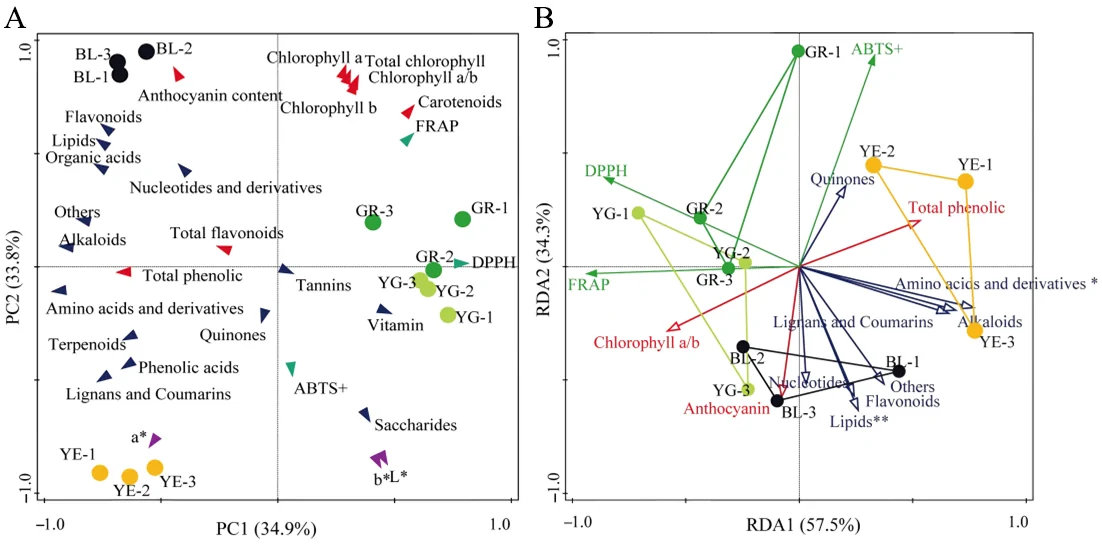
Integrated analysis of metabolic and phenotypic traits in four mung bean varieties. (**A**) PCA of metabolite profiles and physiological parameters. Blue arrows indicate metabolite classes (amino acids, flavonoids, phenolic acids, lipids, terpenoids, alkaloids, organic acids, lignans, coumarins, nucleotides, quinones, tannins, saccharides, vitamins); red arrows indicate bioactive compounds (total flavonoids, total phenolics, anthocyanins) and photosynthetic pigments (chlorophyll a, chlorophyll b, total chlorophyll, carotenoids); purple arrows indicate color parameters (L*, a*, b*). Circles represent variety means with three biological replicates. (**B**) RDA of antioxidant activities constrained by metabolites and color parameters. The RDA model was validated by 999 permutation tests (*p* < 0.05). Asterisks indicate significance at *p* < 0.05 (*) and *p* < 0.01 (**) levels.

PCA showed that PC1 explained 34.9% of the variation on the horizontal axis, while PC2 accounted for 33.8% of the variation on the vertical axis (Figure 8A). The GR and YG form a cluster on the right side of the intersection point of the PCA plot, whereas the BL and YE were away from each other and from the other two varieties. Contents of most metabolites (except tannins, vitamin and saccharides) and bioactive substances (total flavonoids, total phenolic, and anthocyanin) were positively correlated with the BL and YE, but negatively correlated with the GR and YG, while tannins, vitamin and saccharides were positively correlated with the GR and YG. The photosynthetic pigments, including chlorophyll a, chlorophyll b, total chlorophyll and carotenoids were positively correlated with GR, YG and BL, while they were negatively correlated with the YE. Anthocyanin content was positively correlated with the BL and negatively correlated with the YE, GR and YG. In addition, antioxidant abilities, such as DPPH and FRAP, were positively correlated with the GR and YG, while the ABTS^+^ was positively correlated with the YE, but negatively correlated with the BL.

The first and second axes of the RDA explained 57.5% and 34.3% of the total variation, respectively. The amino acids and derivatives total content was the most influential factor followed by the lipids. The total amino acids and derivatives content was significantly negatively correlated with the DPPH and FRAP, and positively correlated with the ABTS^+^. The total lipids content was significantly negatively correlated with the DPPH (Figure 8B).

## 4. Discussion

### 4.1. Core Metabolite Classes and Overall Metabolomic Characteristics of Mung Beans with Different Seed Coat Colors

Metabolomics has been widely applied to investigate seed color formation mechanisms and associated metabolic differences in crops. In sweet sorghum, metabolomic analyses revealed distinct profiles among white, red, and black seeds, with significant differences in flavonoids and anthocyanins [34]. Similar patterns were observed in proso millet [35] and soybean [36]. In cereal grains, integrated metabolomic and transcriptomic analyses demonstrated that flavonoid biosynthesis pathways are closely associated with color formation [37,38]. These studies establish a technical foundation for investigating metabolic differences in differently pigmented mung beans. In this study, metabolomic analysis of four mung bean varieties with distinct seed coat colors identified 1544 metabolites. Amino acids and derivatives, flavonoids, phenolic acids, lipids, and terpenoids were the five most abundant classes with the highest relative signal abundance, collectively accounting for 64.3% of total metabolites. This profile aligns with the functional characteristics of mung beans as high-protein, bioactive-rich foods [1,6]. Amino acids ranked first across all varieties (27.31–31.85%), consistent with the nutritional value of mung bean storage proteins [39]. Flavonoids, as the second-largest class (13.69–20.12%), showed significant inter-varietal differences in subclass composition, reflecting core metabolomic features associated with seed coat color. These results parallel findings in soybeans [36] and other grains [35,38], suggesting that the regulation of secondary metabolism by seed coat color is a conserved biological phenomenon in legumes and cereals.

### 4.2. Analysis on Accumulation Patterns and Functions of Characteristic Metabolites in Mung Beans with Different Seed Coat Colors

The metabolic profiling of four mung bean varieties revealed distinct accumulation patterns that correlate with seed coat color and suggest specialized functional roles. Black-seeded mung bean presented a unique metabolic phenotype, with specific accumulation of flavonoids, anthocyanins, free fatty acids and phenolic acids. Flavonols were the dominant flavonoid subclass in black mung bean, and multiple flavonol glycosides including rutin and baimaside were highly enriched. Rutin has been extensively documented for its anti-inflammatory, antidiabetic, antioxidant, neuroprotective, nephroprotective, and hepatoprotective activities in human studies [40]. Similarly, baimaside (quercetin-3-*O*-glucoside) and other flavonol glycosides exhibit enhanced bioavailability and comparable bioactive properties, including anti-inflammatory, antioxidant and anticancer [41]. The substantial enrichment of these compounds in BL provides a material basis for its potential application in functional food ingredients, but its in vivo health effects need further verification. Anthocyanins are the pivotal substances responsible for the black seed coat phenotype [23]. Multiple anthocyanins and anthocyanidins were significantly upregulated in black mung bean, which is consistent with the pigment accumulation characteristics of other dark-colored coarse cereals such as black soybean and black rice [19,38]. Beyond phenylpropanoid metabolism, BL exhibited significant enrichment in linoleic and α-linolenic acid metabolic pathways (*p* < 0.05), with nine free fatty acids (e.g., TriHOME, EpOME) 3.11–20.07-fold higher and four α-linolenic acid metabolites 2.08–6.01-fold higher, potentially enhancing lipid-soluble antioxidant activity [12]. Free phenolic acids (23.61% of total phenolics) were significantly higher than in YE (19.46%), YG (18.46%), and GR (18.27%), confirming elevated phenolic accumulation in black seeds [11,19].

Yellow-seeded mung bean was characterized by abundant accumulation of amino acids and their derivatives, with eight essential amino acids 1.06–1.39-fold higher than other varieties, with a significantly higher level of essential amino acids than other varieties. Notably, L-methionine in YE and BL was approximately three-fold higher than in YG and GR, addressing the typical sulfur–amino acid deficiency in mung beans [5,39]. Key amino acids (L-histidine and L-valine) were significantly higher than in YG/GR, supporting well the YE’s use as a high-protein variety [5]. In terms of flavonoid metabolism, yellow mung bean was prone to synthesize isoflavones and common flavones, and the isoflavonoid biosynthesis pathway was obviously enriched. Isoflavones such as daidzin, daidzein and prunetin were specifically highly expressed. Daidzin, a major isoflavone in YE, has been shown to act on the mitochondrial monoamine oxidase–aldehyde dehydrogenase pathway, contributing to its antialcohol and metabolic regulatory activities [42]. Meanwhile, prunetin, another upregulated isoflavone in YE, inhibits inflammatory cytokine production by inactivating the TLR4/MyD88 pathway [43]—highlighting the functional diversity of YE-derived isoflavones [27,44]. The isoflavone enrichment in yellow mung beans is analogous to the metabolic specialization observed in yellow soybeans, where isoflavones serve as the primary bioactive compounds contributing to health benefits [45]. Moreover, vitexin, isovitexin and their glycoside derivatives reached the highest content in yellow mung bean, with vitexin-2″-O-rhamnoside identified as a characteristic high-content flavone [46]. Vitexin and isovitexin are ubiquitous dietary flavonoids in edible and medicinal plants. With high safety and multiple pharmacological properties, they exert extensive therapeutic effects on multiple body systems via antioxidant, anti-inflammatory, anticancer, antibacterial and neuroprotective actions [45,46,47,48]. Notably accumulating evidence indicates that mung bean-derived vitexin and isovitexin contribute substantially to the grain’s documented hypoglycemic, hypolipidemic, hepatoprotective, and anti-aging properties, providing a theoretical basis for the development of functional foods and nutraceuticals targeting metabolic syndrome and age-related disorders [49,50,51,52,53]. The differential accumulation of these DAMs could indirectly reflect the genotypic divergence in flavonoid C-glycoside biosynthesis and secondary glycosylation modification pathways among mung bean varieties. Specifically, varieties with higher accumulation of vitexin- and isovitexin-derived glycosides tend to have stronger activity in the synthetic pathway of vitexin and isovitexin aglycones, indicating a greater genetic potential to enrich this class of bioactive flavonoid C-glycosides. Furthermore, lignans and coumarins were also enriched in yellow mung bean, which contribute to seed flavor regulation and anti-inflammatory effects [54].

Compared with black and yellow mung beans, green and yellow-green varieties had lower accumulation of core nutrients and bioactive substances such as amino acids and total flavonoids, while tannins, saccharides and vitamins became their dominant components. Photosynthetic pigments including chlorophyll and carotenoids were most abundant in green mung beans, which corresponds to its green seed coat phenotype. On the whole, green and yellow-green mung beans showed weak metabolic differentiation and a more conservative metabolic network. The metabolic superiority of mung bean over soybean in phenolic and flavonoid accumulation has been documented [55]. This comparative advantage further validates the functional significance of the inter-varietal differences observed herein.

### 4.3. Correlation Between Flavonoid Profiles and Antioxidant Capacity

The contribution of different metabolites to antioxidant activity varied with assay methods, which was related to the different reaction mechanisms of DPPH, ABTS^+^, and FRAP. Anthocyanins and flavonols were the main contributors to FRAP, while isoflavones and amino acids were more closely related to ABTS^+^ scavenging capacity. For DPPH scavenging activity, YG showed the highest activity despite moderate flavonoid and anthocyanin levels, which may be attributed to the synergistic effects of tannins, saccharides and other components, indicating that the antioxidant system of mung bean is jointly regulated by multiple bioactive substances. Inter-varietal differences in flavonoids and anthocyanins were specifically associated with antioxidant capacity, consistent with plant secondary metabolite mechanisms [29,56]. BL’s high anthocyanin and flavonol contents correlated with strong FRAP (398.1 mg TE/kg FW), while YE’s high isoflavone and flavone contents aligned with superior ABTS^+^ scavenging ability (592.4 mg TE/kg FW), suggesting subclass-specific actions against different radicals [57,58]. Although total flavonoid content showed no significant difference in physiological assays (Figure 7E), specific accumulation of flavonols and anthocyanins conferred BL’s antioxidant potential, indicating that subclass composition better reflects activity than total content [34,59]. Correlation analysis confirmed anthocyanins as core contributors to BL’s antioxidant capacity (r > 0.8 with FRAP, *p* < 0.01), paralleling black rice and soybeans [19,60]. YE’s isoflavones and flavones correlated with ABTS^+^ activity (RDA: amino acids positively correlated with ABTS^+^, *p* < 0.05), likely due to phenolic hydroxyl-ABTS^+^ binding [4,16] and luteolin-like antioxidant properties [61]. YG exhibited the highest DPPH activity (297.7 ± 29.7 mg TE/kg FW) despite moderate flavonoid/anthocyanin levels, suggesting synergistic contributions from tannins and carbohydrates, confirming antioxidant system complexity [6,29].

In summary, the metabolomic differences among differentially pigmented mung bean varieties concentrate in flavonoid subclasses, amino acids, and fatty acids, directly regulating antioxidant capacity manifestations. BL’s anthocyanins/flavonols and YE’s isoflavones/amino acids confer distinct functional characteristics, providing key insights for breeding and functional food development. Future transcriptomic integration may elucidate molecular mechanisms of color-regulated metabolite accumulation [36,62]. Further studies incorporating multiple varieties per color class and transcriptomic/genotypic validation are still needed to clarify the independent effect of seed coat color on metabolite accumulation.

## 5. Conclusions

This study presents a comprehensive metabolomic profiling of four mung bean varieties with distinct seed coat colors, demonstrating that seed coat pigmentation is closely associated with metabolic divergence among varieties, and flavonoid subclasses, amino acids, and fatty acids are the main differential metabolites related to seed coat color. Black mung beans (BL) are characterized by significant enrichment of anthocyanins, flavonols, and phenolic acids, correlating with elevated FRAP. In contrast, yellow mung beans (YE) exhibit a unique metabolic profile dominated by essential amino acids and isoflavones, contributing to superior ABTS^+^ scavenging capacity. These findings elucidate the metabolic basis underlying the antioxidant activities of different mung bean varieties. Seed coat color can be used as a preliminary reference indicator for nutritional quality evaluation, providing a theoretical foundation for breeding functionally specialized varieties and developing tailored functional foods.

## Data Availability

The original contributions presented in this study are included in the article/Supplementary Material. Further inquiries can be directed to the corresponding authors.

## Supplementary Materials

The following supporting information can be downloaded at: https://www.mdpi.com/article/10.3390/foods15173020/s1, Table S1: Information for the metabolites identified in mung bean seeds; Table S2: The differentially accumulated metabolites (DAMs) identified from the six comparisons; Table S3: Differentially accumulated flavonoids compounds identified among the four mung bean seeds; Table S4: Differentially accumulated free fatty acids identified among the four mung bean seeds; Table S5: Differentially accumulated phenolic acids identified among the four mung bean seeds; Table S6: Differentially accumulated amino acids and derivatives identified among the four mung bean seeds; Table S7: KEGG enrichment analysis results of DAMs from each comparison.

## Abbreviations

The following abbreviations are used in this manuscript:

ABTS^+^	2,2′-azino-bis(3-ethylbenzothiazoline-6-sulfonic acid)
DAMs	Differentially accumulated metabolites
DPPH	2,2-diphenyl-1-picrylhydrazyl
FC	Fold change
FRAP	Ferric ion reducing antioxidant power
FW	Fresh weight
HCA	Hierarchical cluster analysis
KEGG	Kyoto encyclopedia of genes and genomes
PCA	Principal component analysis
RDA	Redundancy Analysis
TE	Trolox equivalents
UHPLC-QqQ-MS	Ultra-high-performance liquid chromatography coupled with triple quadrupole mass spectrometry
UPLC-MS/MS	Ultra-performance liquid chromatography-tandem mass spectrometry
